# The Strategic Measures for the Industrial Security of Small and Medium Business

**DOI:** 10.1155/2014/614201

**Published:** 2014-05-11

**Authors:** Chang-Moo Lee

**Affiliations:** Department of Police Administration (Criminal Justice), Hannam University, Daejeon 306-791, Republic of Korea

## Abstract

The competitiveness of companies increasingly depends upon whether they possess the cutting-edge or core technology. The technology should be protected from industrial espionage or leakage. A special attention needs to be given to SMB (small and medium business), furthermore, because SMB occupies most of the companies but has serious problems in terms of industrial security. The technology leakages of SMB would account for more than 2/3 of total leakages during last five years. The purpose of this study is, therefore, to analyze the problems of SMB in terms of industrial security and suggest the strategic solutions for SMB in South Korea. The low security awareness and financial difficulties, however, make it difficult for SMB to build the effective security management system which would protect the company from industrial espionage and leakage of its technology. The growing dependence of SMB on network such as internet, in addition, puts the SMB at risk of leaking its technology through hacking or similar ways. It requires new measures to confront and control such a risk. Online security control services and technology deposit system are suggested for such measures.

## 1. Introduction

The development of technology entails the possibility of leakage [1]. South Korea has shown rapid growth in technology during the past 20 years, particularly in IT sector. The remarkable development in technology increased, however, a risk of technology leakage. The leakage of industrial technology presupposes, therefore, high technology and marketability [2–4]. South Korea did not possess high technology to be leaked in 1960s and 1970s when main industries were shoes and textiles.

Since the 1980s, however, South Korea has rapidly been developing in such industries as telecommunication, automobile, ship-building, and electronics. Many companies in these industries have possessed their own cutting-edge technology. The companies with world-class technology have achieved dominant positions in the world market. The Swiss International Institute for Management Development (IMD) evaluated South Korea's technological competitiveness 11th in the world in 2013 [5].

The growing portion of the PCT (patent cooperation treaty) applications would also refer to the technological power which subsequently increases the possibility of technology leakage. South Korea ranked fifth in terms of PCT filing. South Korea filed 11,848 PCT applications in 2012, representing an increase of 13.4% on 2011. This corresponds to 6.1% of all PCT applications filed in the world ([6, Pages 24–27]).

Samsung acquired 4,676 patents in the United States in 2013, the second highest in the world, after IBM (IFI claims patent services, 2014). In addition, ETRI (Electronics and Telecommunications Research Institute) of South Korea was reported to register patents the most in the world in a comprehensive evaluation conducted for research institutes, universities, and government agencies, followed by MIT and Stanford University in the United States [7].

Thus, South Korea has been described as an emerging economic powerhouse with high technologies in many industries. Since such technologies have astronomical economic value, many foreign and domestic companies seem to have a strong interest in the technologies, which might drive them to commit illegal activities such as industrial espionage.

In addition, lots of technology leakage occurs among the small and medium business (SMB) because SMB occupies most of the companies and lacks in the investment of security. The technology leakages of SMB would account for more than 2/3 of total leakages during last five years [8]. Some stronger and strategic measures should be designed for the industrial security of SMB. The purpose of this study is, therefore, to analyze the problems of SMB in terms of industrial security and suggest the strategic solutions for SMB.

## 2. The Problems of the Industrial Security of SMB

Even though the technology leakage of SMB tends to decrease from 15.3% of the companies surveyed in 2008 to 12.1% in 2012, the average loss has been increasing from US $ 901,000 in 2008 to US $ 1,570,000 in 2012, which seemed to result from the leakage of high value technology. Moreover, 37% of SMB suffered from industrial technology leakage more than twice, which shows the loosened security measures of SMB.

On the other hand, the level of security investment of SMB is only 55% of large companies in terms of security products and services. The security expenditures of SMB occupy 11.7% of security products and 19.3% of security services, while most of companies consist of SMB; see Table 2. It shows that the security of SMB remains at a poor stage, which needs to be improved with investing more attention and resources upon security system.

The SMB's problem of security is also shown in the evaluation of security capability. SMB recorded only 58 points while large companies and R&D institutes had 89 and 88 points each in the level of security capability in 2013. The distribution of level clearly indicates the seriousness of the problem, as shown in Table 3. There was no SMB evaluated as excellent, whereas 59.4% of large companies and 45.5% of R&D institutes were evaluated as excellent. More than half of SMB were evaluated as dangerous in the level of security capability.

The sources of the industrial technology leakage show that the leakage of SMB is usually committed by retired employees. The leakage by retired employees is 74.6%, as indicated in Table 5, while it is 9.2% by current employees, 15.1% by vendor, and 15.1% by rival company. The leakage by retired employees continues to increase from 62.4% in 2008 to 74.6% in 2011, while the leakage by current employees tends to decrease from 23.6% in 2008 to 9.2% in 2011. Another significant change comes from rival company. The proportion of rival company increased from 7.9% in 2008 to 15.1% in 2011, in terms of the sources of technology leakage.

As shown in Table 5, the loosened security management (52.7%) was suggested as the major cause of SMB industrial technology leakage, followed by lack of security awareness (46.4%) and individual profit pursuit (30.3%). Complaint on company's treatment (22.4%) and financial difficulty of security investment (22.4%) were listed to be the following major causes of SMB technology leakage; see Table 4.

According to the industrial security management survey report by the small and medium business administration in 2012, in addition, SMB regarded preventing leakage threat from inside as the primary goal for the protection of industrial technology. It was shown that 71.5% of SMB surveyed selected the security efforts against inside threat as the most important one among security measures to be performed. Security awareness (36.5%) and the prevention of key personnel turnover (26.1%) were chosen to be the next important measures for industrial security. Other measures also included building supervision system of security management (21.5%) and acquiring security personnel and equipment (20.8%).

## 3. The Strategic Measures for the Industrial Security of SMB

### 3.1. Providing Online Security Control for SMB

**Figure 1:**
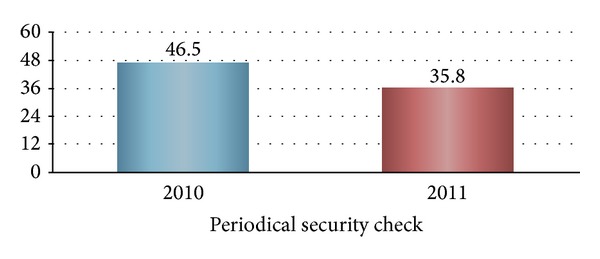
Periodical security check of SMB (%). Source: KISA, information security survey, 2012.

Business work process continues to rely on online network such as internet. It is also inevitable that cutting-edge technology and competitive trade secrets should be stored to computer. The risk of leakage through online network has, therefore, been growing [9]. Most of large companies are likely to employ online security monitoring services for 24 hours a day, 365 days. The high cost of online monitoring services, however, makes it difficult for SMB to use the services, although SMB needs the services. The percentage of SMB experiencing damages from the intrusion of network system was 10.5% of the companies that responded to the Survey of Information Security by Korea Internet and Security Agency (KISA) in 2012. It was shown, in addition, that 63.6% of SMB did not spend any expenditure on information security. Only 1.4% of the SMB surveyed was revealed to have invested more than 10% of the expenditures for informatization to protection of information and technology. The security situation of SMB seems to have worsened. As shown in Figure 1, a periodical security check of SMB decreased from 46.5% in 2010 to 35.8% in 2011.

According to the survey report of the small and medium business administration on industrial security, in addition, 61.5% of SMB respondents answered that the government's online security control services are necessary for industrial security. Most of the companies surveyed also answered to apply for online security control service or consider applying for the service if provided. The 34.5% of the respondents would apply for the service and 50.1% answered to consider it. They also wanted the government to help them build security system with the support of industrial security education and security vulnerability assessment. Such requests would reflect the increasing needs of industrial security for SMB which could not afford to apply online security control services and hire more security personnel due to financial difficulties. It seems to be urgent that most of SMB need to build an effective security management system, which could be materialized with the strong governmental support.

### 3.2. Utilization of Technology Deposit System

Some large companies are trying to steal the cutting-edge technology developed by SMB which usually supplies its products to the large company. Large companies extract the technology by various methods such as demanding a detail drawing of design under the pretense of contract. The SMB would have to suffer such unfair acts by the large company due to its predominant position.

A technology deposit system was, thus, introduced to solve the problem in 2007. According to the technology deposit system, the SMB with core technology or industrial secrets can keep them at reliable public places by which its technology could be protected. When the industrial technology of the SMB is leaked out, the SMB could demonstrate and verify its development of the technology and exclusive rights by showing it stored at the deposit center.

However, a lack of awareness about the technology deposit system restricts its utilization. Only 29.4% of SMB surveyed, as indicated in Table 6, was shown to recognize the deposit system. More than 2/3 of the respondents did not know the presence of the deposit system, which reveals the problem of promotion. In addition, the burden of deposit expenses and one-year contract renewal would hamper the utilization of the system. The expansion of the technology deposit system requires, therefore, the financial support of the government to reimburse SMB for the deposit expenses, along with the active promotion of the system.

## 4. Conclusion

The damage of SMB from the leakage of industrial technology has been ever increasing; see Table 1. The low security awareness and financial difficulties, however, make it difficult for SMB to build the effective security management system which would protect the company from industrial espionage and leakage of its technology. The growing dependence of SMB on network such as internet, furthermore, puts the SMB at risk of leaking its technology through hacking or similar ways. It requires new measures to confront and control such a risk.

Online security control services and technology deposit system are suggested for such measures. These measures could enhance to a certain extent the industrial security of SMB. The low security awareness and financial difficulties seem to be the main obstacles to equip the SMB with such measures. However, these obstacles cannot be removed without a strong governmental support in financial and political ways. The economic competitiveness can be achieved with the balanced development between large companies and SMB. This is why the government should give more attention and resources to SMB to attain the goal.

## Figures and Tables

**Table 1:** The recent damage trends of technology leakage of SMB (2008~2012).

Year	Percentage of technology leakage	Average amount of damage per case (US $)	Number of samples
2008	15.3%	901,000	1,500
2009	14.7%	1,020,000	1,500
2010	13.2%	1,490,000	1,350
2011	12.5%	1,580,000	1,475
2012	12.1%	1,570,000	1,501

Sources: small and medium business administration, *A Report on  Technology Leakage of SMB*, 2013.

**Table 2:** Security expenditures (%).

	Government agencies	Financial institutions	Large companies	Small and medium businesses	Others	Total
Security products	33.4	19.4	**21.3**	**11.7**	14.1	100.0
Security services	28.1	12.9	**32.9**	**19.3**	6.8	100.0

Total	32.6	18.5	**23.0**	**12.8**	13.2	100.0

Source: small and medium business administration, *The Survey Report of the Small and  Medium Business Administration on Industrial Security, *2012 [10].

**Table 3:** Level of security capability (points, %).

	Points	Distribution of level				
		Excellent	Good	Ordinary	Weak	Dangerous
Large companies	89	59.4%	25.0%	15.6%	0.0%	0.0%
Small and medium businesses	**58**	**0.0%**	**17.3%**	**15.4%**	**15.4%**	**51.9%**
R and D institutes	88	45.5%	36.4%	18.2%	0.0%	0.0%

Source: KAIT, industrial technology security issue, 2013, vol. 9.

**Table 4:** Sources of SMB industrial technology leakage (multiple responses, %).

	Retired employees	Current employees	Vendor	Rival company	Others
2008	62.4	23.6	21.0	7.9	5.7
2009	67.0	19	20.4	12.2	3.6
2010	74.5	8.5	10.6	12.8	4.3
2011	74.6	9.2	15.1	15.1	3.2

Source: small and medium business administration,* The Survey Report of the Small and Medium Business Administration on Industrial Security, *2012 [10].

**Table 5:** Main causes of SMB industrial technology leakage (multiple responses, %).

	Loosened security management	Lack of security awareness	Individual profit pursuit	Complaint on treatment	Financial difficulty of security investment
Percentage	52.7	46.4	30.3	22.4	22.4

Source: small and medium business administration, *The Survey Report of the Small and Medium Business Administration on Industrial Security, *2012 [10].

**Table 6:** Awareness of technology deposit system (%).

	Know	Do not know
Manufacturing	32.3	67.7
Service	25.9	74.1
Construction	22.9	77.1

Total	29.4	70.6

Source: Small and medium business administration, *A Report on  Technology Leakage of SMB*, 2013.
